# Lectures on the Theory and Practice of Vaccination

**Published:** 1899-06

**Authors:** 


**Lectures on the Theory and Practice of Vaccination.** By Robert Cory, M.D. Pp. 122. London: Baillière, Tindall and Cox. 1898.

This book contains the substance of the lectures which Dr. Cory periodically delivers to vaccination students. The opening chapter is devoted to the discussion of the necessity for compulsory vaccination; and then follow chapters on the histology of the vaccine and small-pox vesicles, the difference between a primary and secondary vaccination, the eruptions that occasionally follow vaccination, the practical details of vaccination, and the relation of cow-pox to small-pox. The coloured plates and numerous statistical tables give an additional value to a work which undoubtedly should prove of service to the student. Unfortunately the work was written before the final report of the Royal Commission on Vaccination was issued, and consequently no reference is made to those changes in both the medical and lay minds which have led to the recent modification of our vaccination law, and to the adoption of glycerinated lymph. Indeed we find no reference to the bacteriological researches of Pfeiffer, Copeman, and Klein in connection with vaccinia; nor is mention made of the storage of lymph with glycerine, with a view to eliminating extraneous organisms. We should have wished that greater emphasis was laid upon the necessity for rendering the skin aseptic, sterilising the instruments, and the after-treatment of the arm on aseptic principles.

Dr. Cory regards generalised vaccinia as due to wholesale auto-vaccination, and that this is sometimes the case there can be no doubt. At the same time the experiments of Straus,

Chambon, Ménard, and Chauveau all point to the fact that a genuine general infection may follow vaccination—this quite distinct from auto-inoculation. Dr. Cory's book may be found suitable for elementary teaching purposes, but it cannot be regarded as embodying modern ideas in respect to the theory and practice of vaccination; and there is no excuse for the absence of an index.

